# Development of a novel transcription factors-related prognostic signature for serous ovarian cancer

**DOI:** 10.1038/s41598-021-86294-z

**Published:** 2021-03-30

**Authors:** He Li, Nayiyuan Wu, Zhao-Yi Liu, Yong-Chang Chen, Quan Cheng, Jing Wang

**Affiliations:** 1 The Affiliated Cancer Hospital of Xiangya School of Medicine, Central South University/Hunan Cancer Hospital, Changsha, Changsha, 410008 Hunan People’s Republic of China; 2 Department of Neurosurgery, Xiangya Hospital, Central South University, Changsha, 410008 Hunan People’s Republic of China

**Keywords:** Cancer models, Gynaecological cancer

## Abstract

Growing evidence suggest that transcription factors (TFs) play vital roles in serous ovarian cancer (SOC). In the present study, TFs mRNA expression profiles of 564 SOC subjects in the TCGA database, and 70 SOC subjects in the GEO database were screened. A 17-TFs related prognostic signature was constructed using lasso cox regression and validated in the TCGA and GEO cohorts. Consensus clustering analysis was applied to establish a cluster model. The 17-TFs related prognostic signature, risk score and cluster models were effective at accurately distinguishing the overall survival of SOC. Analysis of genomic alterations were used to elaborate on the association between the 17-TFs related prognostic signature and genomic aberrations. The GSEA assay results suggested that there was a significant difference in the inflammatory and immune response pathways between the high-risk and low-risk score groups. The potential immune infiltration, immunotherapy, and chemotherapy responses were analyzed due to the significant difference in the regulation of lymphocyte migration and T cell-mediated cytotoxicity between the two groups. The results indicated that patients with low-risk score were more likely to respond anti-PD-1, etoposide, paclitaxel, and veliparib but not to gemcitabine, doxorubicin, docetaxel, and cisplatin. Also, the prognostic nomogram model revealed that the risk score was a good prognostic indicator for SOC patients. In conclusion, we explored the prognostic values of TFs in SOC and developed a 17-TFs related prognostic signature to predict the survival of SOC patients.

## Introduction

Ovarian cancer (OC) is one of the most lethal gynecological cancer globally, with 295,414 new cases and 184,799 deaths in 2018^1^. The disease encompasses a heterogeneous group of malignancies with epithelial ovarian cancer (EOC) accounting for 90% of all cases. Based on tumor cell histology, EOC is classified as serous, endometrioid, mucinous, clear cell, others, or unspecified. Data shows that more than 50% of EOC is serous ovarian cancer (SOC)^2^. Approximately 60% of OC patients are diagnosed with late-stage disease, partly due to limited test methods. Platinum-based chemotherapy is the main treatment for OC patients; a high proportion of OC patients suffer from platinum resistance, leading to insignificant improvement in the 5-year survival rate in the past decade^3^.

Multiple prognostic biomarkers have been discovered in OC^4^. For instance, CA125 was the firstly identified prognostic biomarker and is used clinically^5,6^. The mutation status of BRCA1/2 is proven to be associated with patient prognosis and chemosensitivity^7^. Several promising prognostic OC biomarkers have been recently discovered such as; VEGF, HE4, mesothelin, M-CSF, osteopontin, kallikrein(s), bikunin, EphA2, and soluble EGF receptor^4^. With the development of multi-omics, numerous genomic variations, miRNAs, lncRNAs, and metabolites have been suggested to serve as prognostic biomarkers^8–13^.

It is difficult to blame OC on a single specific issue due to its heterogeneity^14^. Models integrating multiple genes spring up in OC and other cancers as the global gene expression data becoming more feasible to use. So far, autophagy, DNA repair pathway, and the Notch pathway-related prognostic signatures have been developed for OC^15–17^. Gene methylation panel, miRNAs-based signature, miRNA-lncRNA signature were established because of the vital role of epigenetics in OC prognosis^18–20^. Recently, a novel and non-invasive radiomic prognostic signature was derived based on computed tomography (CT) images, which significantly improved prognostic methods and could be exploited to guide personalized therapy of OC^21^.

Transcription factors participate in various biological processes including cancer proliferation, invasion and migration, cell cycle, apoptosis, EMT, differentiation, and drug resistance^22^; therefore, numerous TFs have been suggested to be potential treatment targets and prognostic biomarkers in various cancers^22,23^. However, it is not clear whether TFs can be utilized to predict the prognosis of SOC. Therefore, we aimed to explore the prognostic values of TFs in SOC and to identify a novel TFs related prognostic signature for SOC.

## Results

### Clinical characteristics of the study patients

**Figure 1:**
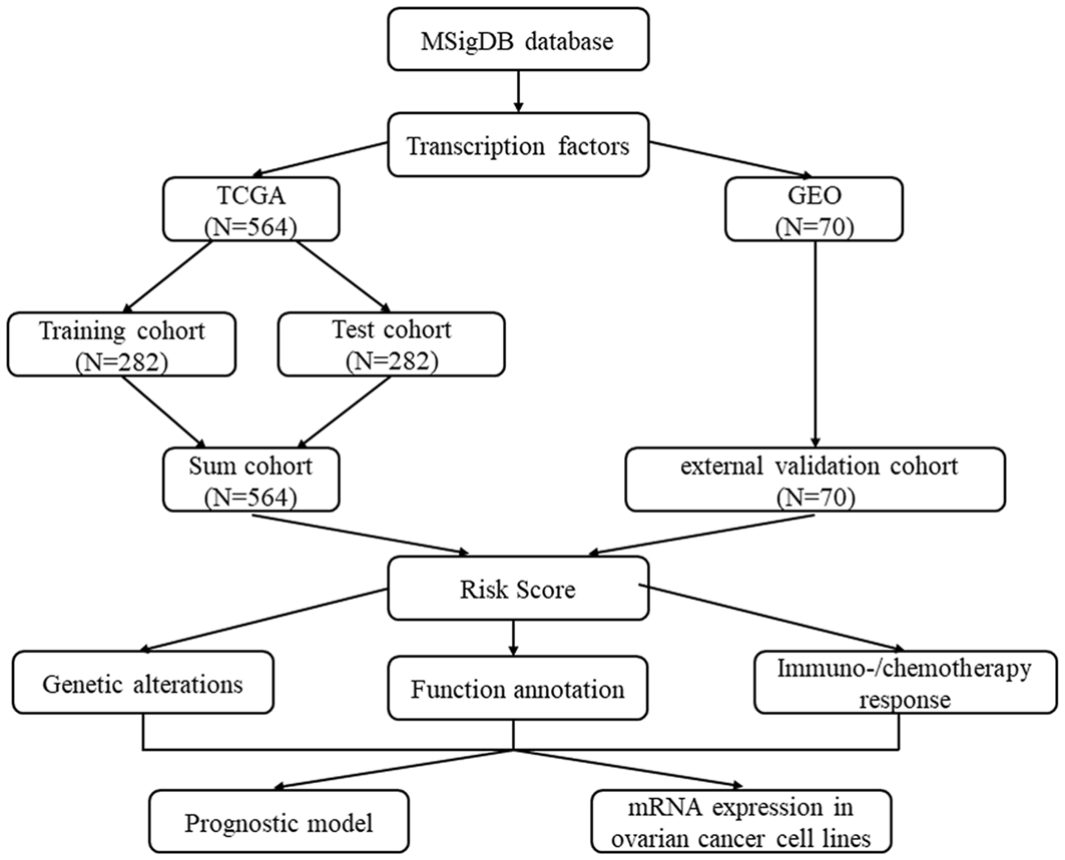
The workflow of this study.

The TFs mRNA expression profiles of 564 SOC subjects in the TCGA database and 70 SOC subjects in the GEO database were screened. All patients from the TCGA database were randomly separated into training (n = 282) and test (n = 282) cohorts and there was no difference between two cohorts in age (*p* = 0.4104), FIGO stage (*p* = 0.6542), Grade (*p* = 0.6890) and living status (*p* = 0.6393) (Supplement Table 2). Patients from the GEO database were used as the external validation cohort. The workflow indicated in Fig. 1 shows the process of the study.

### Construction and validation of TFs-related risk signature for SOC

**Figure 2:**
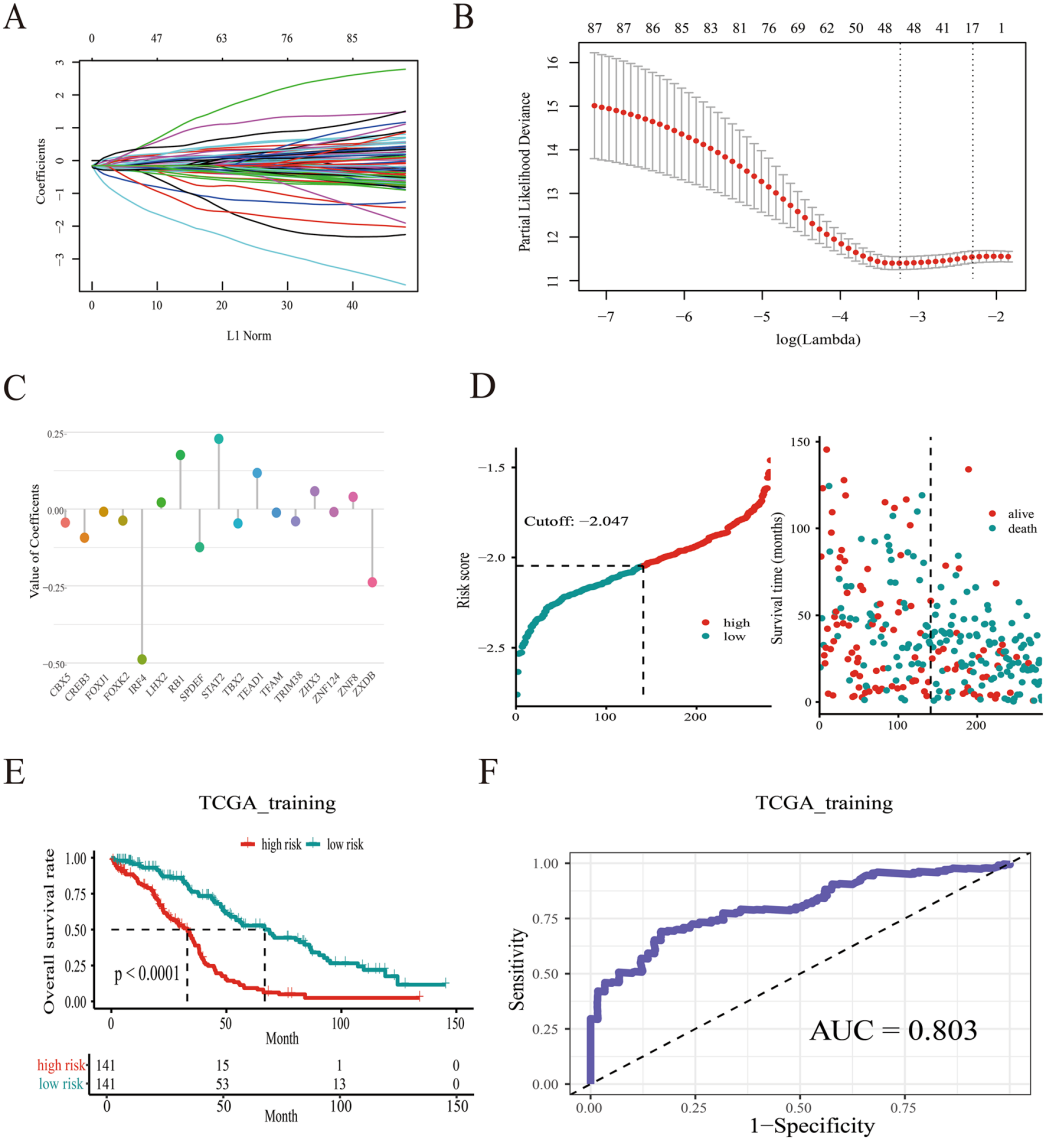
A 17-TFs related prognostic signature was established in TCGA training cohort. (**A**–**C**) The lasso coefficients of the 17 TFs for overall survival in TCGA training cohort; (**D**) The distribution of risk score and alive status in TCGA training cohort; (**E**,**F**) The 17-TF related prognostic signature predicts overall survival in SOC and the prediction performance was evaluated by calibration curve.

**Figure 3:**
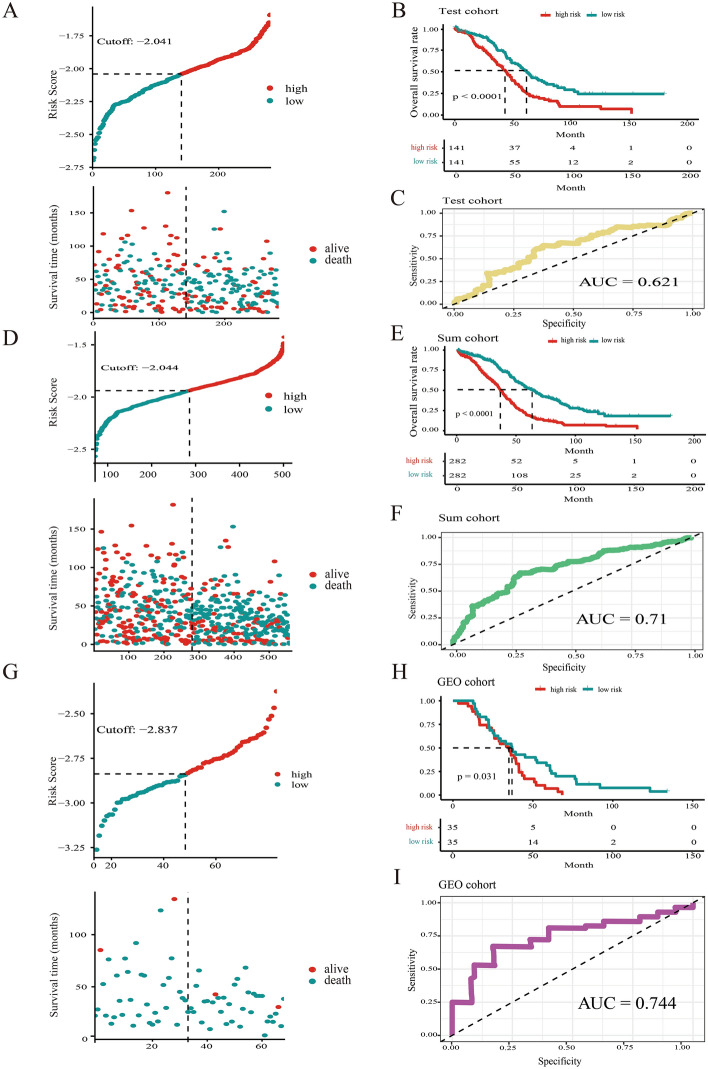
The 17-TFs related prognostic signature was verified in TCGA test cohort (**A**–**C**), TCGA sum cohort (**D**–**F**) and GEO cohort (**G**–**I**).

All the TFs derived from MSigDB database were analyzed by using univariate cox regression analysis. 88 TFs associated with overall survival of SOC patients were selected (Supplement Table 3). To narrow down the panel and optimize gene assembly, the 88 selected gene were employed in the LASSO cox regression model, in which we obtained 17 TFs (CBX5, CREB3, FOXJ1, FOXK2, IRF4, LHX2, RB1, SPDEF, STAT2, TBX2, TEAD1, TFAM, TRIM38, ZHX3, ZNF124, ZNF8 and ZXDB) (Fig. 2A,B). Their coefficients were listed in Fig. 2C and were substituted into formula to calculated the risk score. Based on the median risk value as a cutoff point (cutoff =  − 2.047), patients were divided into high-risk and low-risk groups (Fig. 2D). There was a significant difference in prognosis between high-risk group and low-risk group (Fig. 2E). The prediction feature was assessed by the ROC curve and the AUC value of 0.803 (Fig. 2F). Similarly, patients with high-risk scores had a poor OS in the test cohort, sum cohort, and external validation cohort (*p* < 0.0001, *p* < 0.0001, *p* = 0.031, respectively, Fig. 3A–I). It is worth mentioning that there was a significant difference in OS rate between high- risk score group and low-risk score group in ICGC cohort and Tothill cohort (*p* = 0.035, *p* = 0.005, respectively; Supplement Fig. 1A-1B). Moreover, the distribution of six molecular subtypes identified by Tothill et al., were different in high-risk group and low-risk group (*p* < 0.001, Supplement Fig. 1C). All these findings indicated that there was a significant association between the TFs-related risk signature and SOC patients’ prognosis and it might be used to accurately predict the outcome of patients with SOC.

**Figure 4:**
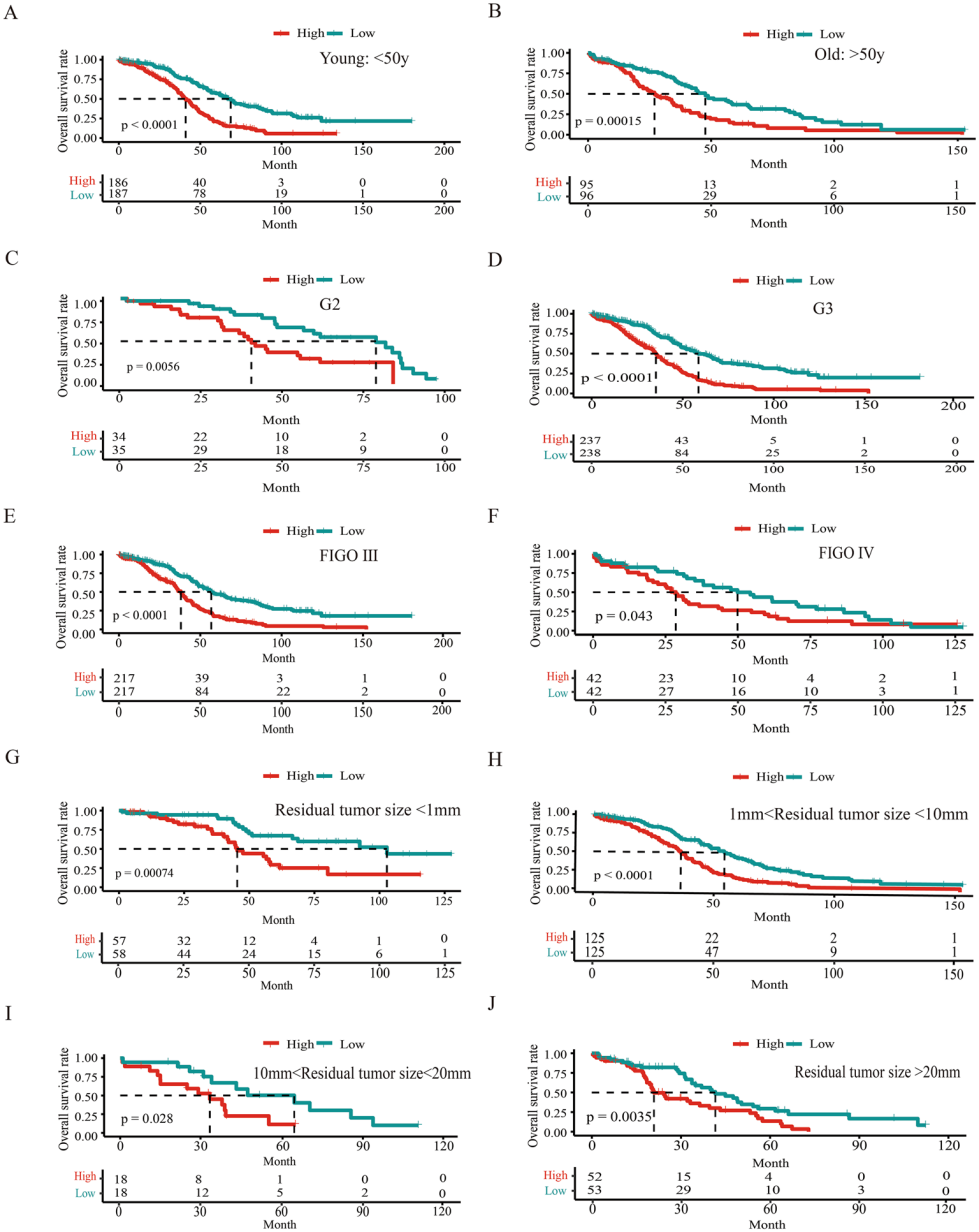
Construct a stratified analysis based on age (**A**,**B**), grade (**C**,**D**), FIGO grade (**E**,**F**) and residual resection status (**G**–**J**) to analyze the association between the TFs related prognostic signature and overall survival.

The association between the risk signature and various clinicopathologic features including grade, FIGO stage, and residual tumor size was analyzed. The results showed that there was no difference in risk score among different grades (*p* > 0.05, Supplement Fig. 2A). Patients with FIGO IV and III had higher risk scores compared with patients with FIGO I and II, (*p* < 0.01, Supplement Fig. 2B). There was also a significant association between residual tumor size and risk score (*p* < 0.001, Supplement Fig. 2C). A stratified analysis based on age, grade, FIGO grade, and residual tumor size was constructed. The high-risk group had shorter OS than the low-risk group in all these stratified cohorts, indicating that the TFs-related risk signature could be used to accurately distinguish the prognosis of SOC patients, without considering the clinical parameters (*p* < 0.01, Fig. 4A–J).

### Association between the TFs-related risk signature and genetic alterations

**Figure 5:**
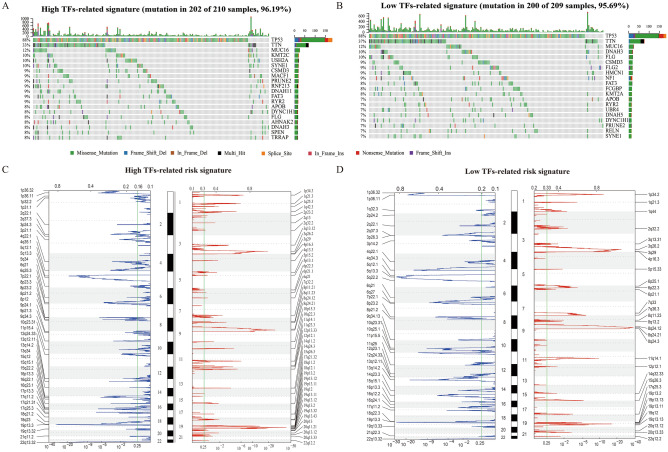
Genetic alterations including somatic mutations (**A**,**B**) and SCNAs (**C**,**D**) assigned to high-risk group (**A**,**C**) and low-risk group (**B**,**D**); The green line represents the significance threshold (*q* value = 0.25).

Genetic alterations including somatic mutations and SCNAs were identified in the lowest risk (1st) and highest risk (4th) groups. There was no difference between the two groups in the mutation frequency of TP53, TIN, MUC16, DNH3, CSMD3, FAT3, FLG, and APOB (Fig. 5A,B). Patients in the lowest risk group had a higher probability of somatic mutation in the DNAH3 gene, while a higher mutation frequency of KMT2C and USH2A occurred in the highest risk group (Fig. 5A,B). We also analyzed the SCNAs between both groups (Fig. 5C,D). The G values between the highest-risk and lowest risk groups were significantly different, although the amplification (including 19q.12, 8q24.21, 3q36.2 and 11q14.1) and focal peaks (including 19p13.3, 22q13.32, 5q12.1, and 17q11.2) were detected in both groups. Additionally, several regions of amplifications harboring multiple oncogenes, such as 8q24.21 (PTV1, MYC)19q13.2 (NFKBIB), 19p13.12 (NOTCH3) and 15q26.3 (IGF1R) were only detected in the highest risk group. Moreover, focal deletion peaks were detected in the group with high-risk scores such as 9p21.3 (CDKN2A, CDKN2B) and 10q23.31 (FAS) (Fig. 5C,D).

### Consensus clustering analysis to construct the cluster model

**Figure 6:**
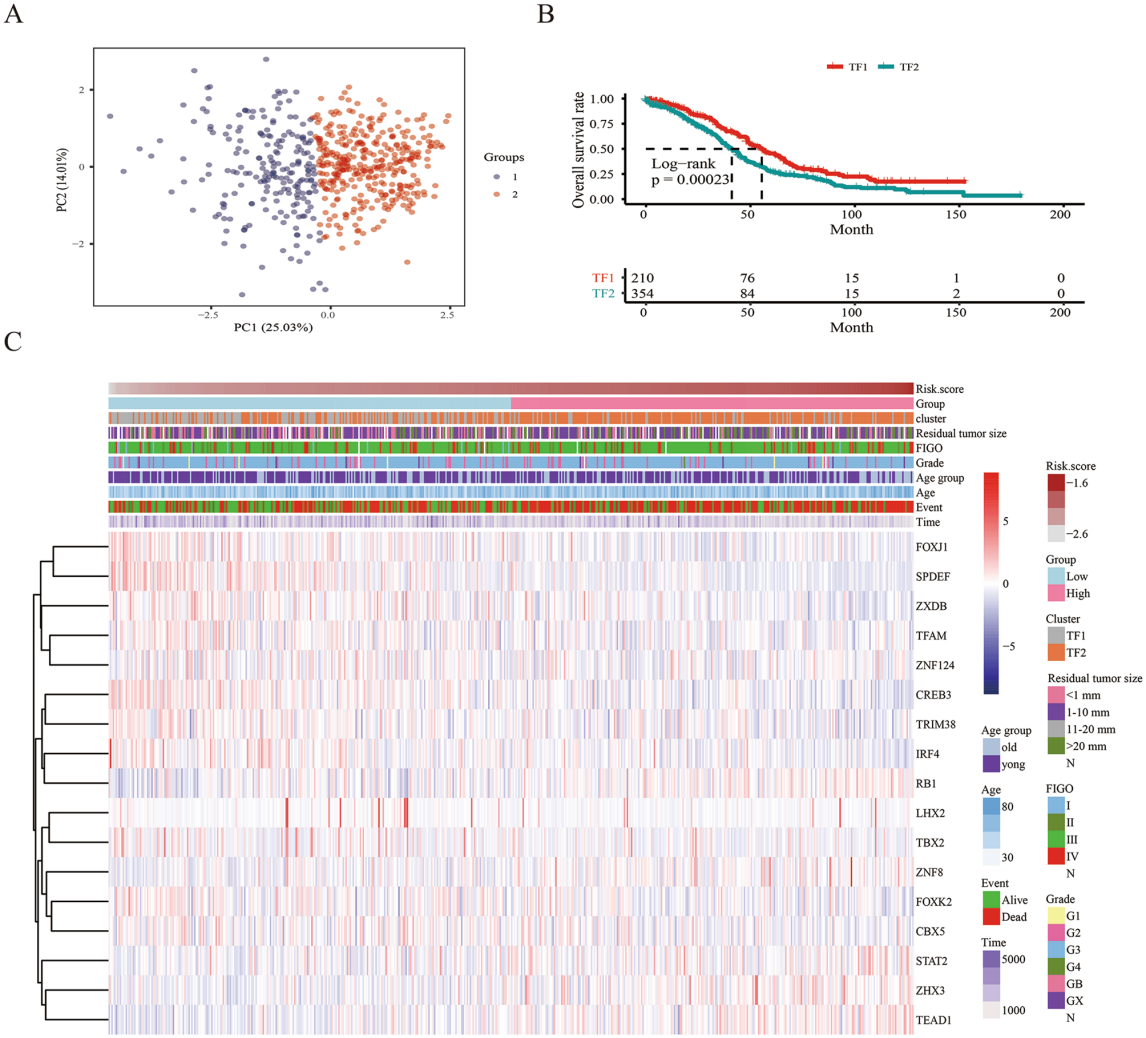
The significant difference of the mRNA expression of the 17 TFs (**A**) and overall survival (**B**) between the two clusters (TF1 vs. TF2); (**C**) The relationship among TFs mRNA expression, risk score, alive status, overall survival, age, FIGO sage, Grade and residual resection status.

All SOC samples from the TCGA database were divided into different clusters using consensus clustering analysis; two was found to be the optimal number of clusters (Supplement Fig. 3A-2B). The principal component analysis (PCA) explored the significant difference in mRNA expression of the 17 TFs between the two clusters (TF1 vs. TF2) (Fig. 6A). There was a significant difference in OS between the two clusters (*p* = 0.00023, Fig. 6B). The TF1 cluster was related to a lower risk score, while it had no association with age, FIGO stage, grade, and residual tumor size (Fig. 6B,C).

### Pathways enrichment analysis to identify the biological function of TFs in SOC

**Figure 7:**
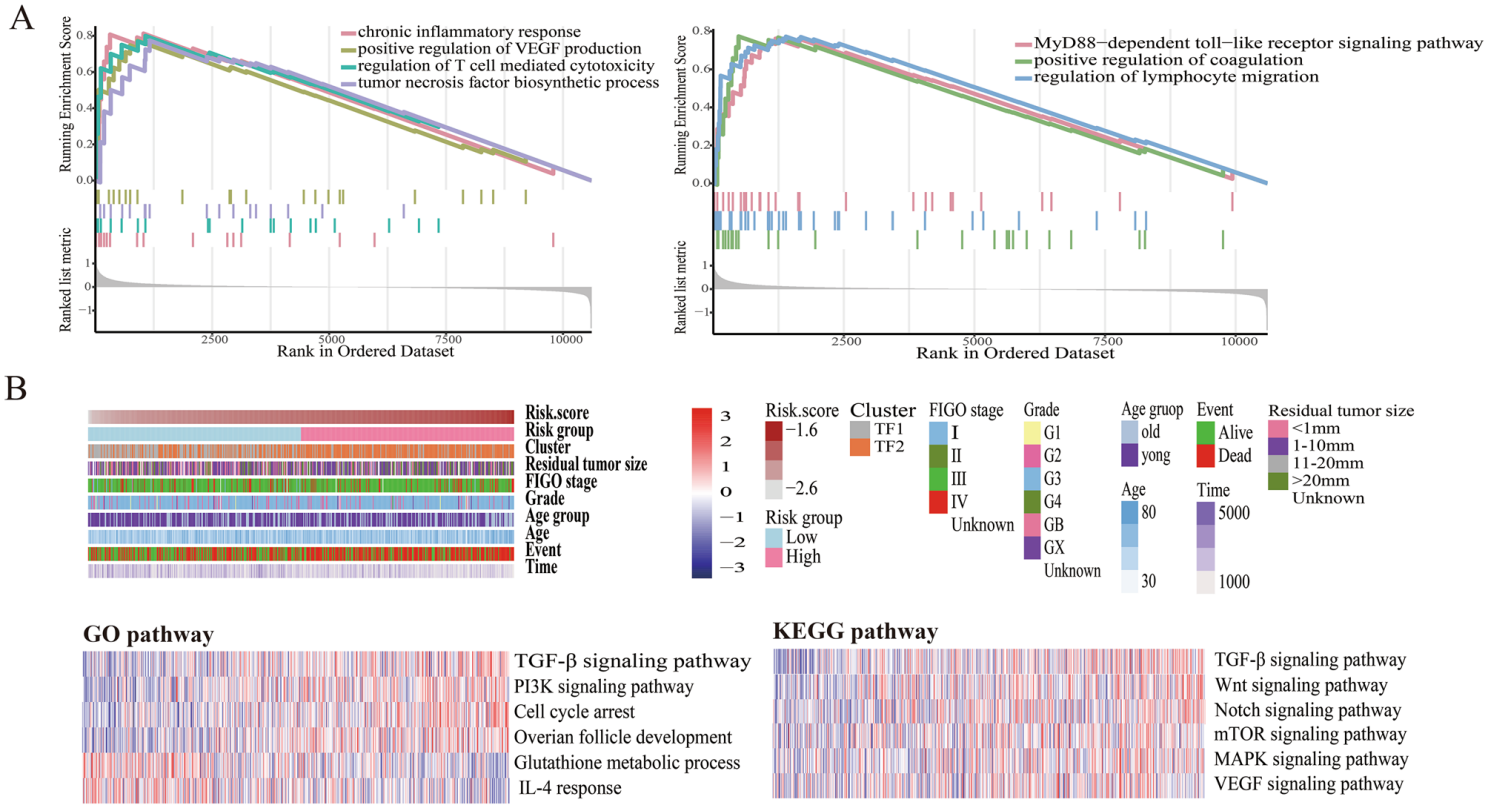
Functional annotation of TFs related prognostic signature. (**A**) GSEA assay to identify the different pathways between high-risk score group and low-risk score group; (**B**) GSVA assay to explore the distribution of risk scores and clinical features (upper), and gene set enrichment of different pathways in GO terms and KEGG pathways (lower).

First, GSEA analysis was performed to identify the differences in biology function among patients with high-risk and low-risk scores. The results indicated differences in several pathways including chronic inflammatory response, positive regulation of vascular endothelial growth factor, regulation of T cell-mediated cytotoxicity, tumor necrosis factor biosynthetic process, MyD88-dependent toll-like receptor signaling pathway, positive regulation of coagulation, and regulation of lymphocyte migration (Fig. 7A). Then, GSVA analysis was executed to reveal the functional enrichment status of the 17-TFs in SOC. Signal pathways with a high correlation coefficient and statistical significance were selected from GO gene sets and KEGG pathway analysis. The higher gene set enrichment scores of all the selected signaling pathways were associated with higher risk scores, except the glutathione metabolic process and IL-4 response pathways. Some pathways, including the TGF-β signaling and IL-4 response, were mainly related to the inflammatory response (Fig. 7B). Cell proliferation, invasion, and migration-related pathways including; PI3K signaling, cell cycle arrest, Wnt signaling, Notch signaling, mTOR signaling, MAPK signaling, and VEGF signaling, were also enhanced in the high-risk score group. The biological processes involved in each TF were also analyzed, indicating that they had different biological functions in SOC (supplement Fig. 4A-4B).

### Difference of the potential immune infiltration, immunotherapy and chemotherapy response between high-risk score and low-risk score groups

**Figure 8:**
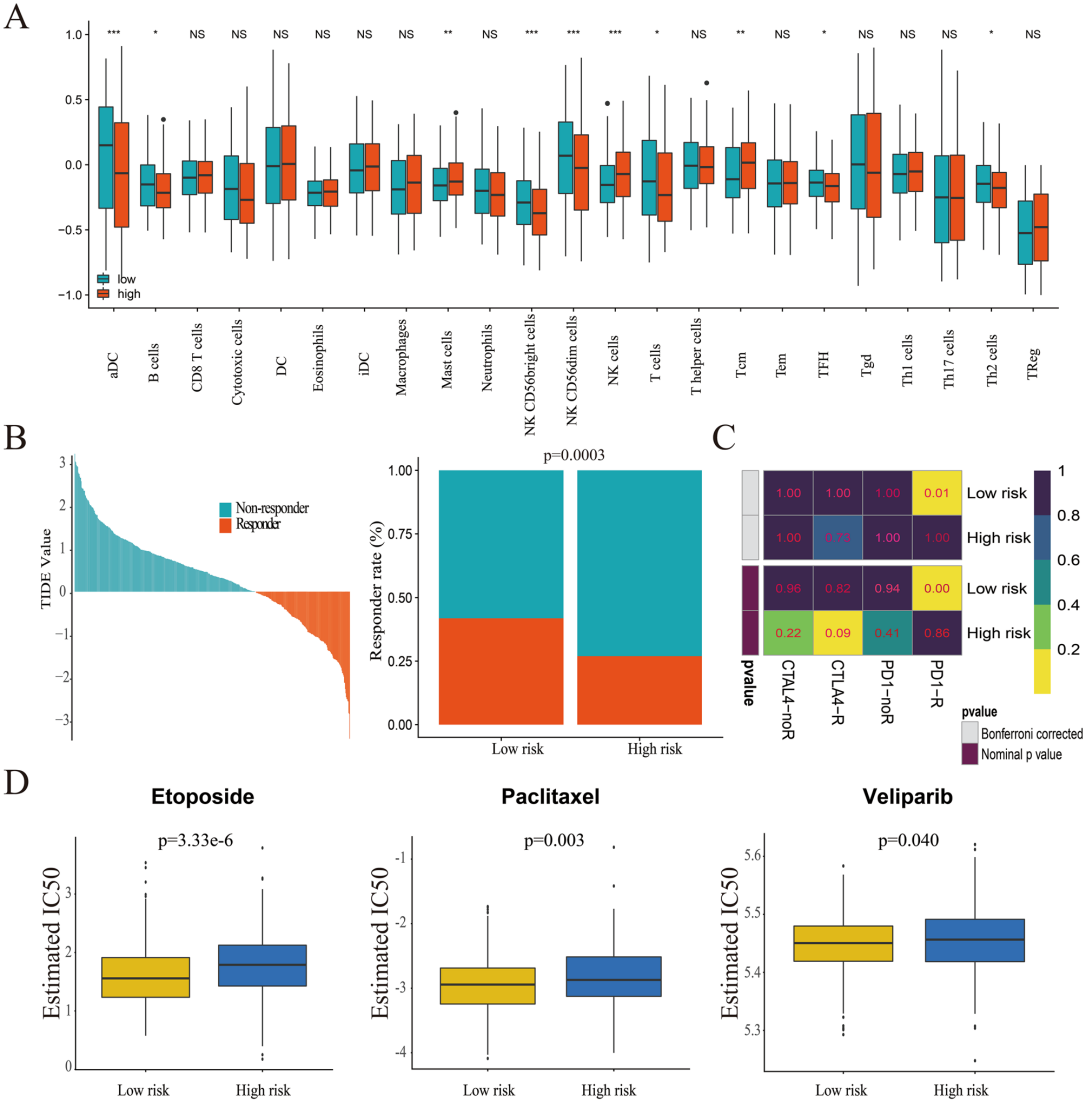
Difference between high-risk score group and low-risk score group in immune infiltration, immunotherapy and chemotherapy response prediction. (**A**) immune infiltration proportion of TIICs between high-risk score group and low-risk score group (*p < 0.05; **p < 0.01; ***p < 0.001); (**B**) the TIDE value and response to immunotherapy of patients with SOC; (**C**) the response to anti-CTAL4 and anti-PD-1 therapy of patients with SOC in both groups; (**D**) Estimated IC50 values indicate the efficiency of chemotherapy by etoposide, paclitaxel and veliparib in both groups.

There was a significant difference between the high-risk and the low-risk score groups in the regulation of lymphocyte migration and T cell-mediated cytotoxicity. Therefore, the potential immune infiltration, immunotherapy, and chemotherapy responses were analyzed. Data showed that the distribution of aDC, B cells, NK CD56bright cells, NK CD56dim cells, T cells, TFH and Th2 cells was lower in the high-risk than the low-risk score group, whereas mast cells, NK cells, and Tcm cells infiltration was higher in the high-risk than the low-risk score group (Fig. 8A). The difference in immune infiltration between two clusters (TF1 vs. TF2) was also determined (supplement Fig. 5). The potential response to immunotherapy for each patient was assessed using the TIDE algorithm. The results suggest that patients with low-risk scores are more sensitive to immunotherapy than those with high-risk scores (*p* = 0.0003, Fig. 8B). Subsequently, the response of anti-CTL4 and anti-PD-1 therapy were explored; patients in the low-risk score group were more likely to respond to anti-PD-1 therapy (*p* = 0.01, Fig. 8C).

Chemotherapy is the main treatment method for SOC and it plays a key role in patients’ survival. The different response to chemotherapy between the high-risk and low-risk score groups was noted. The cell line data obtained from GDSC database were used to train the predictive model and the IC_50_ values for a total of 137 commonly used anti-tumor drugs were determined; the values were calculated by the predictive model. The results indicated that there was a significant difference in the estimated IC_50_ of twenty-one dugs between the high-risk and low-risk score groups (Supplement Table 4). Patients with low-risk scores showed better response to etoposide, paclitaxel, and veliparib (*p* = 3.33*e* − 6; *p* = 0.003; *p* = 0.040, respectively, Fig. 8D). However, the response to gemcitabine, doxorubicin, docetaxel, and cisplatin was similar for patients in the two groups (*p* = 0.077, *p* = 0.131, *p* = 0.169, *p* = 0.182, respectively; Supplement Fig. 6). The different response to all the 137 drugs commonly used to treating cancers in high risk and low risk group were listed in Supplement Table 4.

### Modeling prognostic nomogram for overall survival

**Figure 9:**
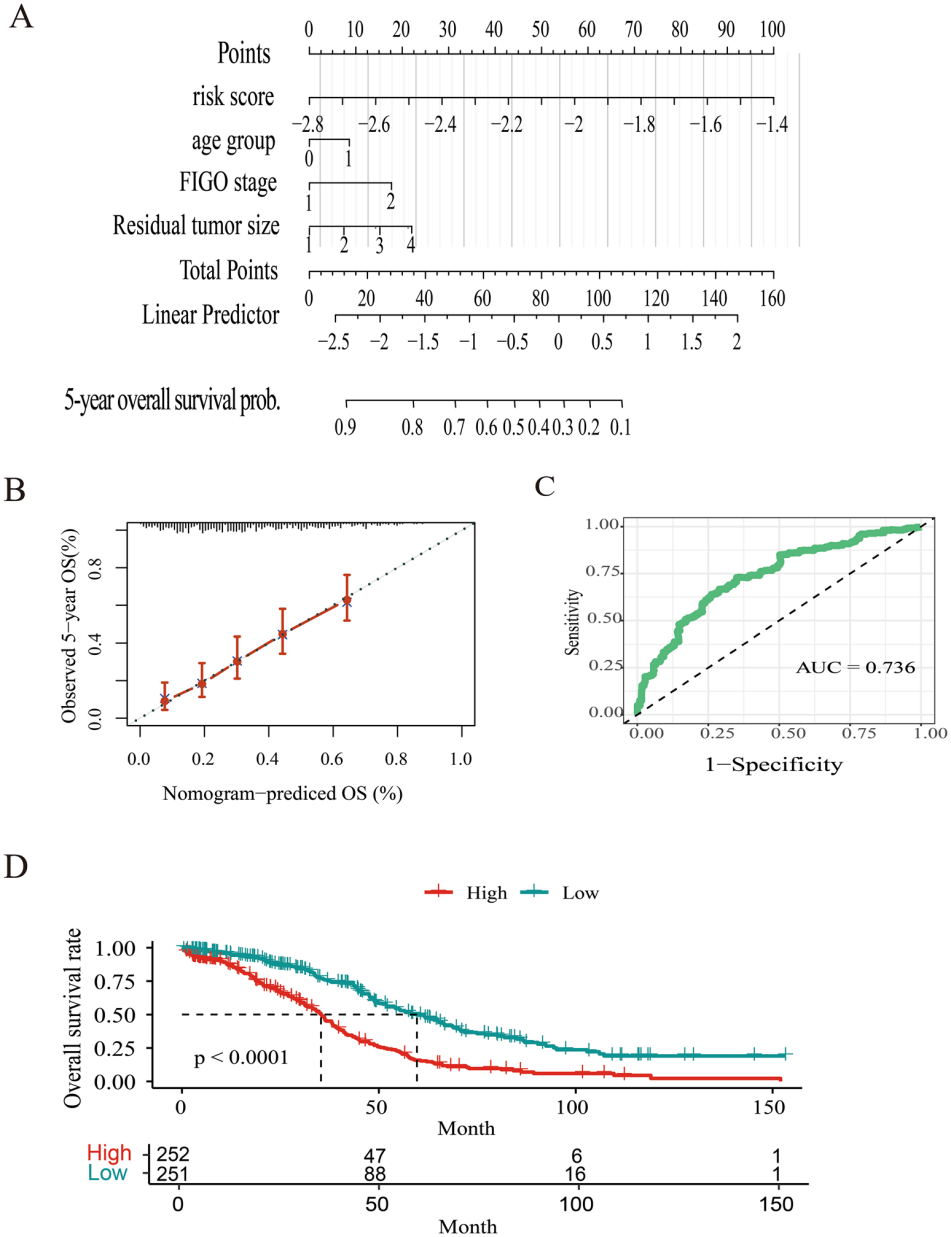
Prognostic nomogram for predicting the 5-year overall survival of SOC patients. (**A**) Prognostic nomogram for glioma patients was created based on clinical risk factors; For age group, 0 means < 50 years old and 1 means > 50 years old. For FIGO stage, 1 means FIGO I + II and 2 means FIGO III + IV. For residual tumor size, 1 means residual tumor size < 1 mm; 2 means 1 mm < residual tumor size < 10 mm; 3 means 10 mm < residual tumor size < 20 mm and 4 means residual tumor size > 20 mm. (**B**) The calibration curve of 5-year overall survival. The predicted probability of overall survival is plotted on the x-axis and the observed OS is plotted on the y-axis; (**C**) ROC curve from the nomogram of 5-year overall survival; (**D**) Comparison of overall survival between two groups divided by the median value of the prognostic model (Low vs. High).

Due to the loss of clinical data, only 503 SOC patients were included in modeling prognostic nomogram for overall survival. First, the independent prognostic factors were identified using univariate and multivariant cox regression (Table 1). The results indicated that risk score (*p* < 0.001, HR = 10.0841), residual tumor size (1–10 mm: *p* < 0.01, HR = 1.5889; 10–20 mm: *p* = 0.008, HR = 2.0247; > 20 mm: *p* < 0.001, HR = 2.0447), and age (*p* = 0.02, HR = 0.7586) were all independent prognostic factor for 5-year overall survival. The FIGO stage was adopted as one of the independent factors given that the FIGO III + IV parameters were statistically significant in univariant cox regression (*p* = 0.03, HR = 2.884), and showed marginal statistical difference in multivariant cox regression (*p* = 0.133, HR = 1.7485). There was no association between grade, stage, and 5-year overall survival of patients. Therefore, risk score, residual disease, age, and FIGO stage were used to construct the nomogram, and each variable was assigned a score on the corresponding axis. The probabilities of the outcomes were determined by the location of the total score on the survival axes, which was calculated by adding up all the variables (Fig. 9A). The nomogram-predicted 5-year overall survival closely corresponded with the observations (Fig. 9B). Calibration curves were used to evaluate the accuracy of the nomogram with an AUC value of 0.736 (Fig. 9C). The prognostic model value was calculated according to prognostic factors and regression coefficient. Patients were divided into two groups by setting the median prognostic value as cutoff; there was a significant difference in the 5-year overall survival rate between the groups (*p* < 0.001, Fig. 9D).

**Table 1:** Univariable and multivariate cox regression analysis of factors affecting overall survival of SOC patients.

Variables	Univariable analysis		Multivariate analysis	
	HR (95% CI)	*p* value	HR (95% CI)	*p* value
Risk score	13.895 (7.637–25.281)	< 0.001***	10.0841 (5.4247–18.7454)	< 0.001***
Residual disease (1–10 mm)	2.069 (1.471–2.912)	< 0.001***	1.5889 (1.1173–2.2596)	< 0.01**
Residual disease (10–20 mm)	2.3 (1.389–3.809)	< 0.001***	2.0247 (1.2066–3.3974)	0.008**
Residual disease (> 20 mm)	2.829 (1.925–4.157)	< 0.001***	2.0447 (1.3745–3.0415)	< 0.001***
Age (< 50 year)	0.646 (0.514–0.813)	< 0.001***	0.7586 (0.6009–0.9578)	0.020*
FIGO stage (III + IV)	2.884 (1.428–5.823)	0.003**	1.7485 (0.8437–3.6237)	0.133
Grade stage (III + IV)	1.297 (0.929–1.81)	0.126	1.0583 (0.7465–1.5003)	0.750

HR, Hazard Ratio; CI, Confidence Interval.

* *p* < 0.05; **p < 0.01; ***p < 0.001.

## Discussion

Ovarian cancer has the highest mortality rate among gynecological cancers. It is reported that more than 90% of OC patients suffer from the EOC subtype. Based on tumor cell histology, EOC is classified as serous (> 50% of cases), endometrioid, mucinous, clear cell, and others or unspecified. Approximately 60% of OC patients are diagnosed with late-stage disease due to the lack of early and effective test methods, leading to poor clinical outcomes. Moreover, the 5-year survival rate has marginally improved in the past decade. Transcription factors play vital roles in various biological processes including tumorigenesis and drug resistance, and may serve as biomarkers to predict the prognosis of SOC.

In this study, a 17-TFs related prognostic signature and cluster model was introduced to predict the clinical outcome of SOC. In detail, 564 SOC patients from the TCGA database were randomly divided into the training and test cohorts. The 70 SOC patients from GEO were deemed as the external validation set. A 17-TFs related prognostic signature was obtained in the training cohort by successively performing univariant cox regression and Lasso cox regression. The 5-year overall survival rate was significantly different between the high-risk and low-risk score groups. The 17-TFs related prognostic signature showed high prediction performance with a high AUC value (AUC = 0.803), and this was validated in the test, sum, and GEO cohorts. Genomic alterations assay results showed that the risk score of SOC patients was associated with genomic aberrations. Besides, the cluster model was constructed using consensus clustering analysis. Consensus clustering analysis is a method for evaluating two questions: how many groups are present in a dataset, and what is the confidence in the number of groups and the group memberships. It provides quantitative and visual ‘stability’ evidence derived from repeated subsampling and clustering and is popular in cancer research. It can not only used in class discovery but also in identification of disease subtype, immune subtype and molecular subtype. What’s more, it might also be used in identify new therapeutic targets of disease. It is worth mentioning that consensus clustering analysis is commonly used in processing single-cell RNA-seq data.

Function annotation was performed using GSEA and GSVA analysis. The GSVA results revealed that the prognostic TFs were mainly related to the TGF-β, PI3K, mTOR, MAPK, VEGF and Wnt signaling pathways, which played important roles in cell proliferation, invasion, and migration. The TGF-β signaling pathway, which participates in immune response, was screened from both GO gene sets and the KEGG pathway. The GSEA results indicated that there were significant differences in the chronic inflammatory and immune responses between the high-risk and low-risk score groups. The difference of potential lymphocyte migration, immune infiltration, immunotherapy, and chemotherapy response between the high-risk score and low-risk score groups were explored due to the big difference in regulating T cell-mediated cytotoxicity. The distribution of several immune cells including T cells, B cells, and NK cells were found to be different between the two groups. Moreover, patients with low-risk scores showed higher response to immunotherapy, especially anti-PD-1 therapy, and chemotherapy drugs including etoposide, paclitaxel, and veliparib. Finally, a nomogram model was constructed combined with clinical data and risk scores. The prediction utility was very high with an AUC value of 0.71 in the TCGA cohort and an AUC value of 0.744 in the GEO cohort. Multiple gene signatures have been developed to predict overall survival, recurrence, and platinum sensitivity in OC. Some gene signatures were also used to predict the overall survival outcome in SOC^15,24,25^. Liu et al. developed a seven gene-based novel signature to predict clinical outcomes of High-Grade IIIc Serous Ovarian Carcinoma with an AUC of 0.715 in the TCGA cohort^24^. A genomic rearrangement signature was constructed in High-Grade Serous Ovarian Cancer (HGSOC) with an AUC of 0.63 in the TCGA cohort^25^. A novel autophagy-related prognostic signature was exploited for SOC with an AUC of 0.703^15^. Compared with the mentioned gene signatures, the 17-TFs related prognostic signature showed basically consistent prediction capacities.

TFs play important roles in various biological processes including cancer proliferation, invasion and migration, cell cycle, apoptosis, EMT, differentiation, and drug resistance in OC. The 17 TFs, except FOXK2, LHX2, SPDEF, TRIM38, ZHX3, and ZXDB, were reported to be involved in the malignant phenotype of OC. The CBX5, a member of chromobox (CBX) family, was suggested to function as a prognostic biomarker and potential target for various cancers; including gastric cancer, breast cancer, and lung cancer^26–29^. The expression of CBX5 was elevated in gastric cancer tumor tissues and it promoted cell proliferation, migration, and invasion^27^. It was highly expressed in breast cancer tissues and correlated to relapse-free survival (RFS) for the patients^28^. Besides, CBX5 was identified as a potential target in regulating lung cancer survival and the stem-like properties of lung CD133^+^-tumor stem-like cells (TSLCs)^29^. It was reported that BRD4 suppression enhanced the cytotoxicity of CHK1 inhibition in HGSOC, which was mediated by the increased expression of CBX5^30^. The CREB3 marker was identified as an oncogene that plays an important role in tumor metastasis in various cancers; including prostate cancer, breast cancer, and osteosarcoma^31–33^. It is reported that the CREB3 antigen may serve as an effective biomarker with high diagnostic sensitivity and specificity in OC, although its prognostic role is unclear^34^. The FOXK2 and FOXJ1 are members of the forkhead box (FOX) transcription factors. Growing evidence shows that FOXK2 participates in a range of key processes in cancer cells, such as cell proliferation, invasion and migration, DNA damage, and autophagy^35^. The function of FOXK2 is context and tumor-specific, and it might serve as an oncogene and tumor suppressor in different cancers^36^. It was reported that FOXK2 inhibited cell proliferation and invasion in breast cancer, non-small cell lung cancer (NSCLC), clear-cell renal cell carcinoma, gastric cancer, and indicated favorable prognosis^37–40^. However, it promoted cell growth and indicated unfavorable prognosis in colorectal cancer and hepatocellular carcinoma^41,42^. The role of FOXK2 in OC is currently unknown. Meanwhile, FOXJ1, negatively regulated by NANOG, exerts anti-cancer proliferation, migration, and invasion activity in OC^43^. Mainly expressed in plasma cells, IRF4 was reported to be associated with tumor stage, histological grade, and completeness of chemotherapy^44^. Multiple evidence has shown that TEAD1 and TFAM are oncogenes in OC, which is concordant with our results^45,46^. The role of ZNF124 in OC is little known, but it was found to inhibit apoptotic death and function as an oncogene in hematopoietic cells^47^. The pRB protein, coded by the RB1 gene, is a famed tumor suppressor in OC, which is similar to our results, and was related to acquired chemotherapy resistance and OC recurrence^48,49^. The ectopic expression of TBX2 is associated with conferred resistance to platinum-based chemotherapy, indicating that it may serve as a predictive marker of the efficacy of platinum-based chemotherapy for patients with OC^50^. Recently, STAT2 was identified to be highly expressed in OC, and the patients involved had poor OS and PFS. It was reported that STAT2 functions as an oncogene by increasing the expression of EGFR^51^. Although single biomarker for prognosis has been widely reported, it is difficult to accurately predict the prognosis of OC patients due to its heterogeneity. As regards the prognostic signature or nomogram, if we can put it into clinical practice in the future, we may be able to identify SOC patients with high-risk of cancer-related death before treatment, and recommend a more aggressive therapeutic strategies with dynamic surveillance.

This study has some limitations: (1) Venous and lymphatic invasion were not considered because the data was missing for most patients. (2) The sample size in the external validation set was small and the predictive ability of the 17-TFs related prognostic signature was not validated in the samples.

In conclusion, we obtained a TFs related prognostic signature and constructed a clinical model for predicting prognosis of SOC patients, which was able to effectively distinguish high-risk populations from the TCGA and GEO datasets.

## Methods

### Datasets and preprocessing

Gene expression profiles and corresponding clinical information were downloaded from the TCGA data source (https://xena.ucsc.edu) and the GEO data portal (https://www.ncbi.nlm.nih.gov/geo/). All TCGA samples were randomly divided into two equal cohort: training cohort (n = 282) and validation cohort (n = 282). The total TCGA data (n = 564) were used to validate as sum cohort. The GEO cohort (n = 70) was used as the external validation set. The patients in ICGC cohort (https://daco.icgc.org/) (n = 115) and Tothill cohort (GSE9899, n = 278) were utilized to explore the association between OS rate and risk score. The clinical characteristics of all subjects in TCGA database were listed in Supplement Table 1. All eukaryotic TFs were downloaded from MSigDB (http://software.broadinstitute.org/gsea/msigdb/genefamilies.jsp).

### L1-penalized (Lasso) cox regression

A robust likelihood-based survival modeling was applied to reduce the dimension and obtain robust survival-associated TFs. The Lasso regression was used to identify the TFs with independent prognostic value. Through 5000 cross-validations, a set of prognostic genes and their LASSO coefficients were obtained according to the highest lambda value, using the Lasso method (lambda 1st) with tenfold cross-validation. Based on the LASSO coefficients, we established a TFs-related prognostics risk score model^52^. The association between risk score, TFs expression and clinical characters was illustrated by heat maps.

### Time-dependent Receiver operating characteristic (ROC)

Time-dependent ROC curve and area under the curve (AUC) were applied to evaluate the prediction accuracy of the five-year prognostic model in the training, test, sum, and GEO cohorts. Besides, it was also used to estimate the prediction performance of the nomogram. All the time-dependent ROC curves were calculated by SurvivalROC package and drew by ggplot2 package.

### Overall survival analysis

The SOC patients were divided into high-risk and low-risk score groups according to the median TFs related prognostic signature or median TFs mRNA expression. Survival curves were illustrated by the Kaplan–Meier method with the long-rank test. The overall survival rate of high and low clusters was also calculated. Furthermore, stratified survival analyses were performed based on different ages, FIGO stage, tumor grade, and residual tumor size.

### Genetic alterations analysis

Genetic alterations including somatic mutations and somatic copy number alterations (SCNAs) were investigated. All the samples obtained from the TCGA database were divided into four groups based on the values of the TFs-related prognostic signature and risk score. The Top 20 genes with the highest mutation frequency in the lowest and highest group were selected. The SCNAs of both groups were identified using the Genomic Identification of Significant Targets in Cancer 2.0 (GISTIC)^53^.

### Gene set enrichment analysis (GSEA)

The GSEA annotation was adopted using the “clusterProfiler” package, to identify potential differences in biological function between SOC patients with high-risk and low-risk scores^54^.

### Consensus clustering analysis

Samples were divided into different clusters using consensus clustering analysis with the R package “ConsensusClusterPlus”^55^. The subsampling parameter was 80% with 1000 times and k (the number of clusters) ranged from 2 to 10. The optimal number of clusters was selected based on cumulative distribution function plots and consensus matrices^56^. Clinical features and different expressions of TFs between clusters were visualized by heat maps.

### Gene set variation analysis (GSVA)

The 17-TFs signature for each SOC sample was established using gene sets enrichment analysis (GSVA). The heat map was used to illustrate the correlation between the 17-TFs, their mRNA expression and the clinical features of SOC. The GSVA package was utilized to reveal the functional enrichment status of these TFs in SOC. The cutoff of the Correlation coefficient was set as 0.5 (*p* < 0.05)^57^. Gene Ontology (GO) terms, Kyoto Encyclopedia of Genes and Genomes (KEGG) pathways were obtained from MSigDB^58^.

### Immune infiltration, immunotherapy and chemotherapy response prediction

The infiltration of 22 subtypes of tumor-infiltrating immune cells (TIICs) was calculated by CIBERSORT algorithm (http://cibersort.stanford.edu/), which is a method for characterizing cell composition of complex tissues from their gene expression profiles^59^.

Tumor Immune Disfunction and Exclusion (TIDE) algorithm (http://tide.dfci.harvard.edu/) is a computational method to accurately outcome of patients treating with immune checkpoint blockade (ICB)^60^. To better understand the immune response function of TFs in SOC, TIDE was employed to estimate the individual likelihood of responding to immunotherapy. There are currently no approved immune therapies for ovarian cancer. Therefore, the response to anti-CTAL-4 or anti-PD-1 therapy was analyzed based on the treatment results of melanoma patients in the subclass mapping method (SubMap, http://www.broad.mit.edu/genepattern/) ^61^.

The chemotherapy response of each patient was evaluated using the Genomics of Drug Sensitivity in Cancer database (GDSC, https://www.cancerrxgene.org). Drugs that are used for SOC treatment were selected for assessment. The half-maximal inhibitory concentration (IC_50_) was calculated and represented the drug response. The R package ‘pRRopheticRredic’ was used with tenfold cross-validation and other parameters by default^62^.

### Prognostic Model construction based on clinical features and risk score

Univariate cox proportional hazard regression analysis was performed to select risk factors (*p* < 0.05). A multivariate COX model based on the selected features and the nomogram chart was constructed using the “RMS” in R software to predict 5-year overall survival^63^. The accuracy of the risk model was assessed using the calibration curve and AUC. Finally, the risk score value was calculated according to the formula, risk score = *β1X1* + *β2X2* + ⋯ + *βnXn* (*β,* regression coefficient; *X*, prognostic factors).

### Statistical analysis

All statistical analyses were performed using R software (version 3·5·3). Significant quantitative differences between groups were analyzed using the two‐tailed Students’ t-test, whereas differences among groups were analyzed using the one‐way ANOVA. The chi-square test was used to analyze the clinical features of different cohorts. Kaplan–Meier method with log-rank test was used to calculate the overall survival rate of SOC patients. Univariate and multivariate Cox regression analyses were performed to evaluate the prognostic value of the risk score and clinical features. The partition around medoids (PAM) algorithm was conducted in the consensus clustering analysis. The correlation between two variables was analyzed with Spearman rank (*p* < 0.05).

## Supplementary Information

Supplementary Information
