# Supplementary material for: Development of a novel transcription factors-related prognostic signature for serous ovarian cancer
Source: Sci Rep. 2021 Mar 30;11:7207. doi: 10.1038/s41598-021-86294-z (PMC8010122; doi:10.1038/s41598-021-86294-z)
Supplement: Supplementary file 1 — Supplementary Information [file 41598_2021_86294_MOESM1_ESM.pdf]

Development of a novel transcription factors-related prognostic signature for serous ovarian cancer

He Li[1], Nayiyuan Wu[1], Zhao-Yi Liu[1], Yong-Chang Chen[1], Quan Cheng[2*], Jing Wang[1*]

[1] The Affiliated Cancer Hospital of Xiangya School of Medicine, Central South University/Hunan Cancer Hospital, Changsha, Changsha 410008, Hunan, P. R. China

[2] Department of Neurosurgery, Xiangya Hospital, Central South University, Changsha 410008, Hunan, P. R. China

[*] Equal contribution

Correspondence to: Jing Wang, email: wangjing0081@hnca.org.cn; Quan Cheng, email: chengquan@csu.edu.cn

Supplement materials

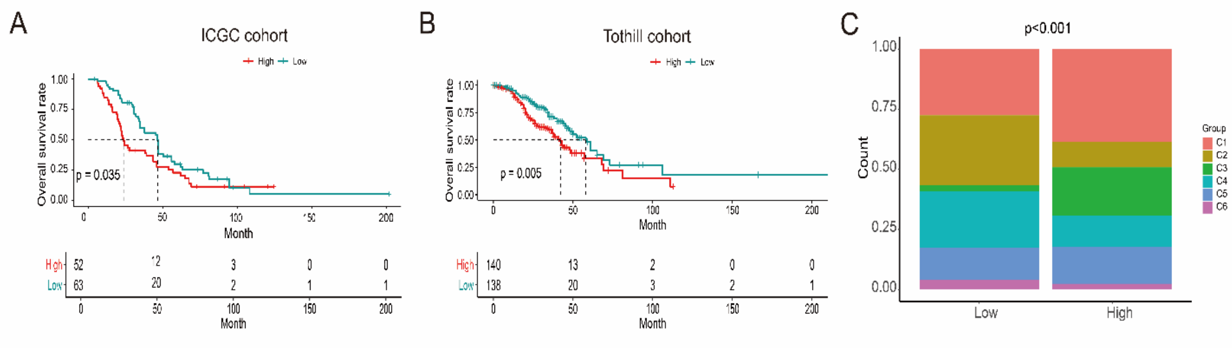

Supplement Figure 1. The association between OS rate and risk score in ICGC cohort (A) and Tothill cohort (B); The distribution of six molecular subtype of ovarian cancer in high-risk group and low-risk group.(C).

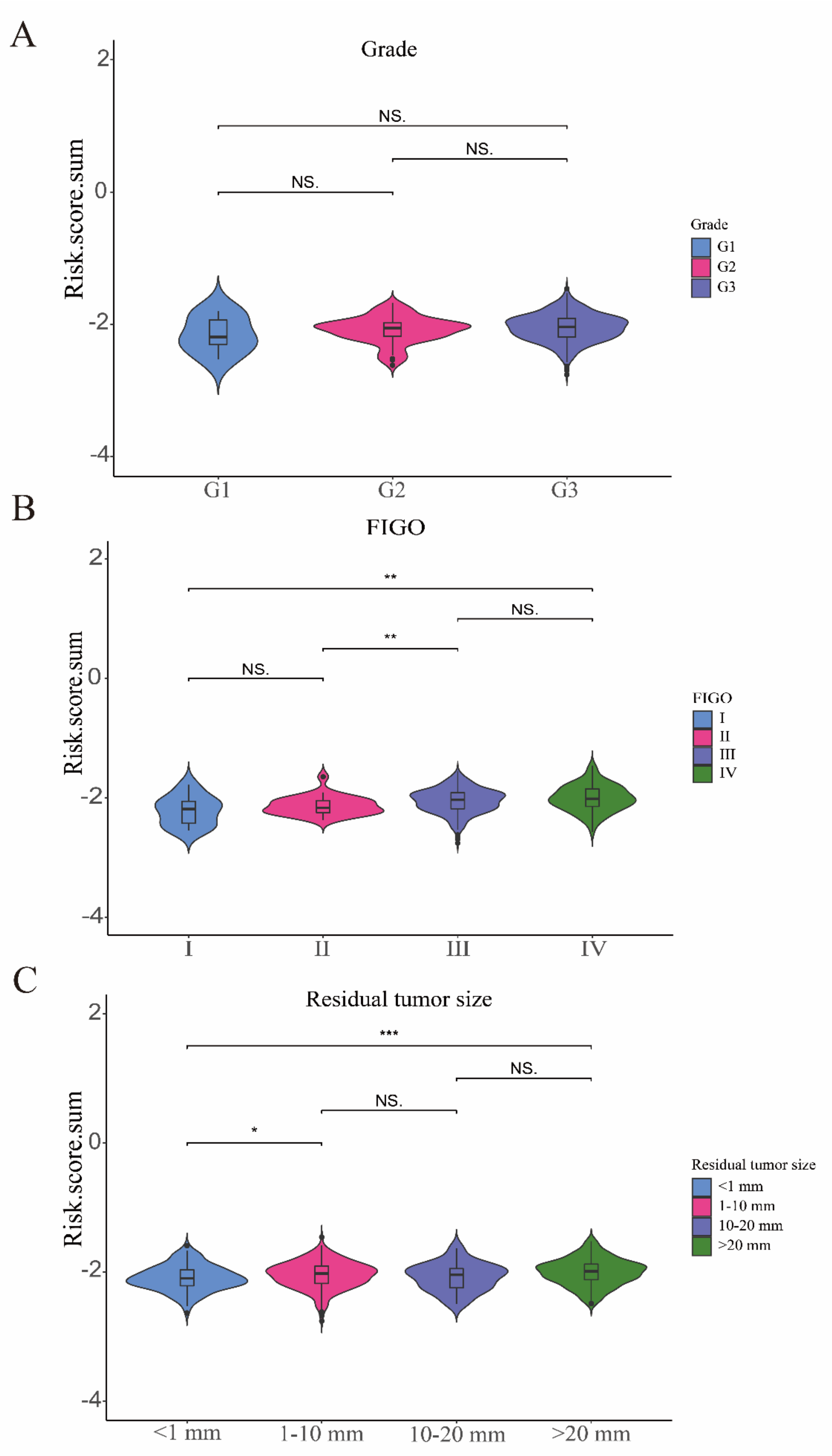

Supplement Figure 2. The association between the risk signature and various clinicopathologic features. (A) Grade; (B) FIGO stage; (C) Residual tumor size

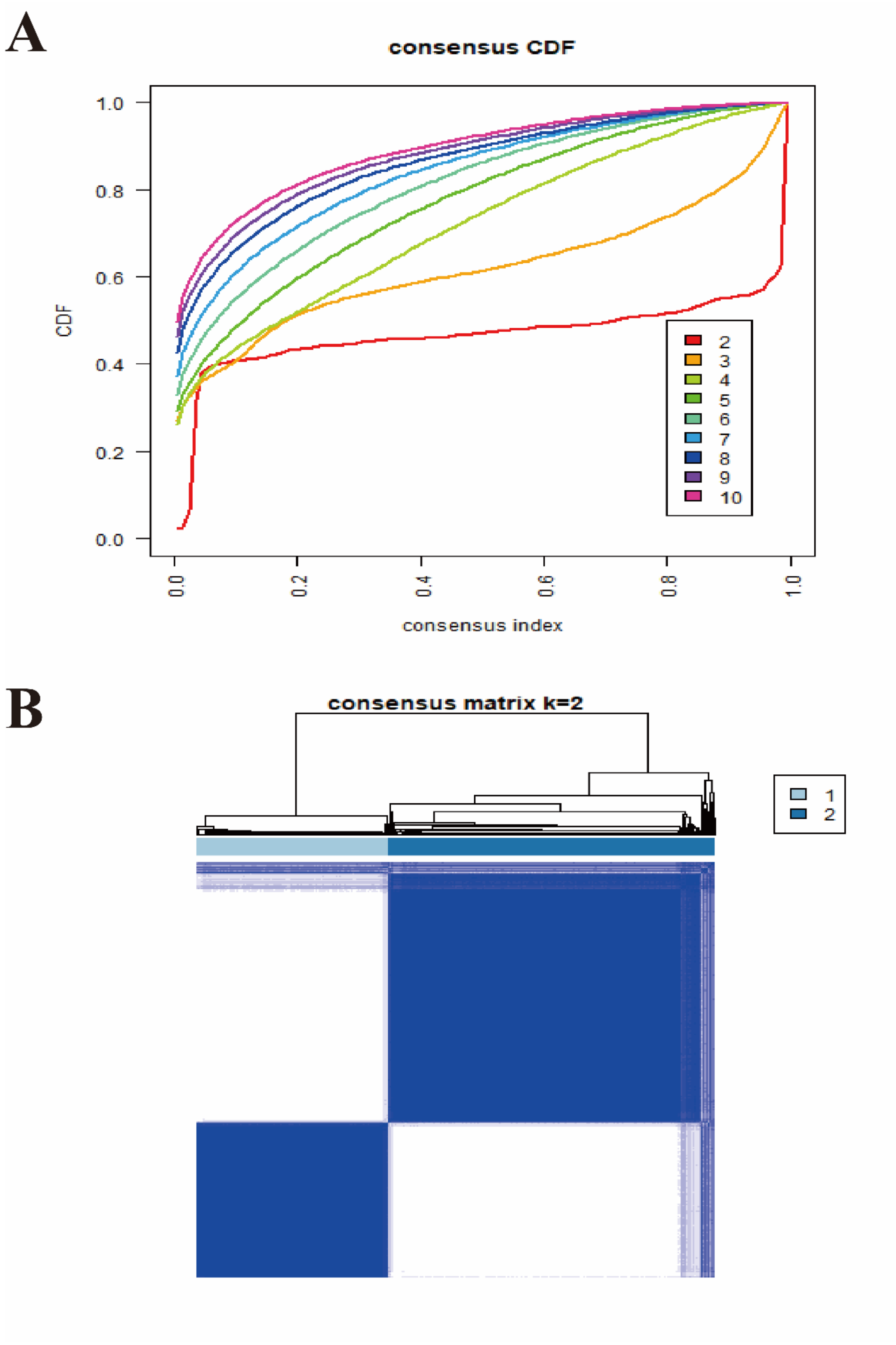

Supplement Figure 3. (A) Clustering by the consensus clustering algorithm with k= 2 to 10; (B) Consensus matrix for 2 clusters.

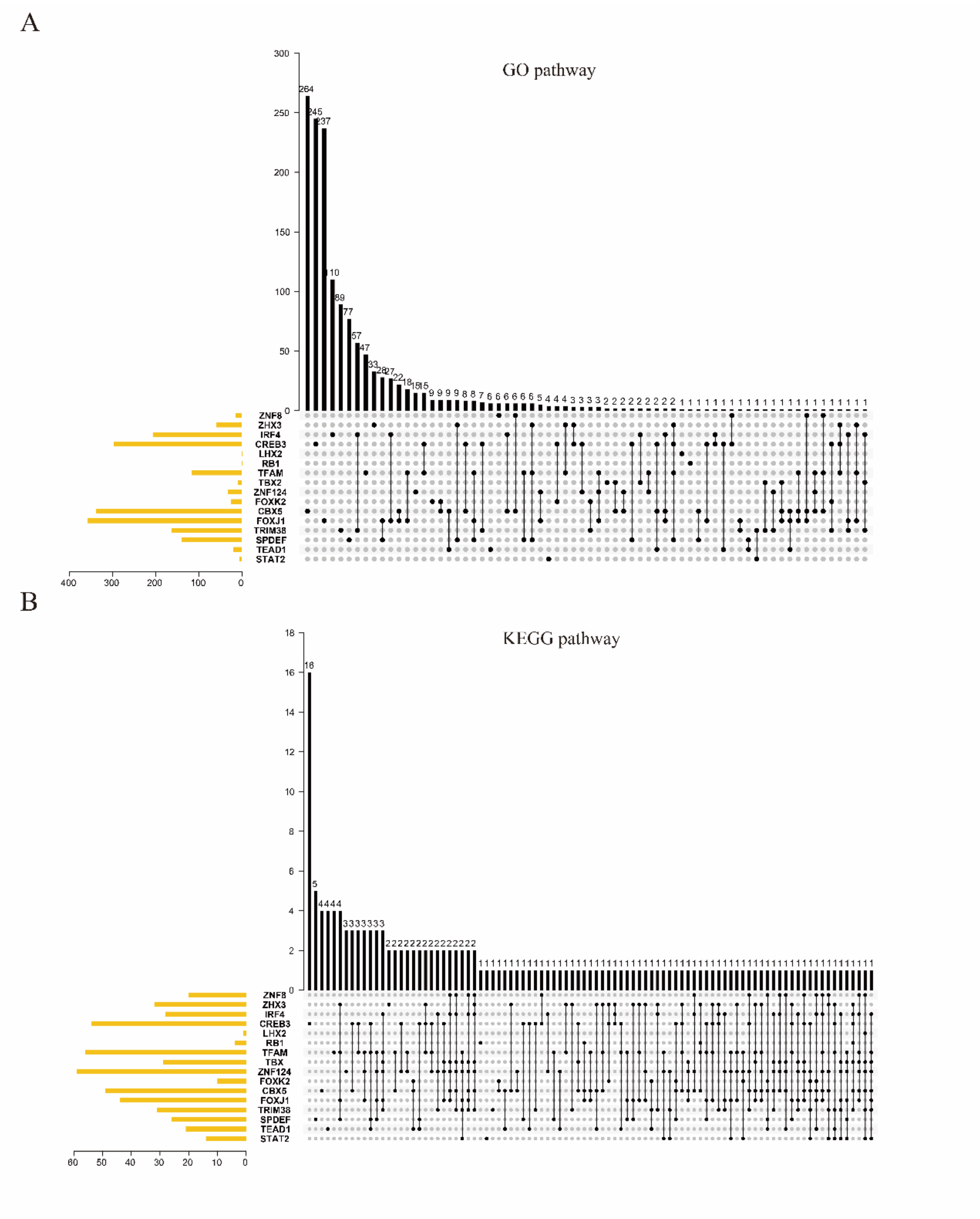

Supplement Figure 4. Venn diagram of biological processes involved in each of the 17 TFs from GO pathway (A) and KEGG pathway (B).

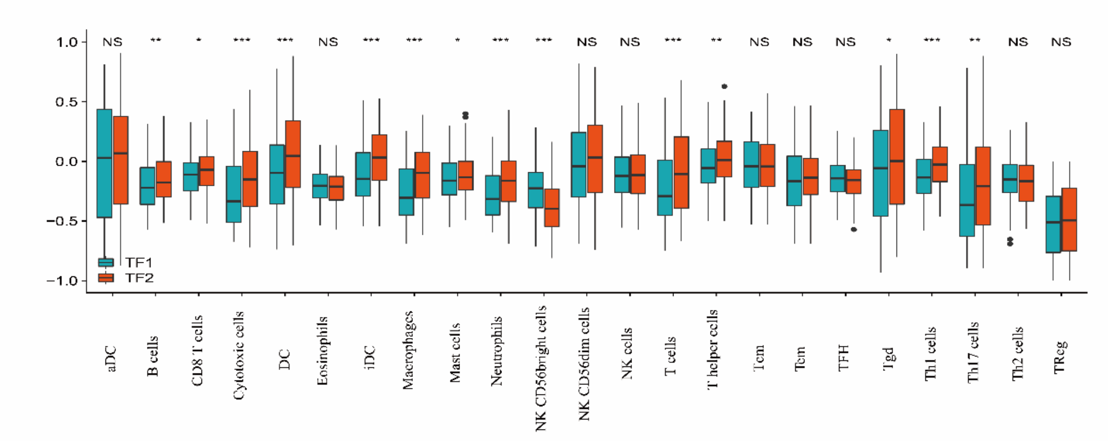

Supplement Figure 5. Immune infiltration proportion of TIICs between two clusters.

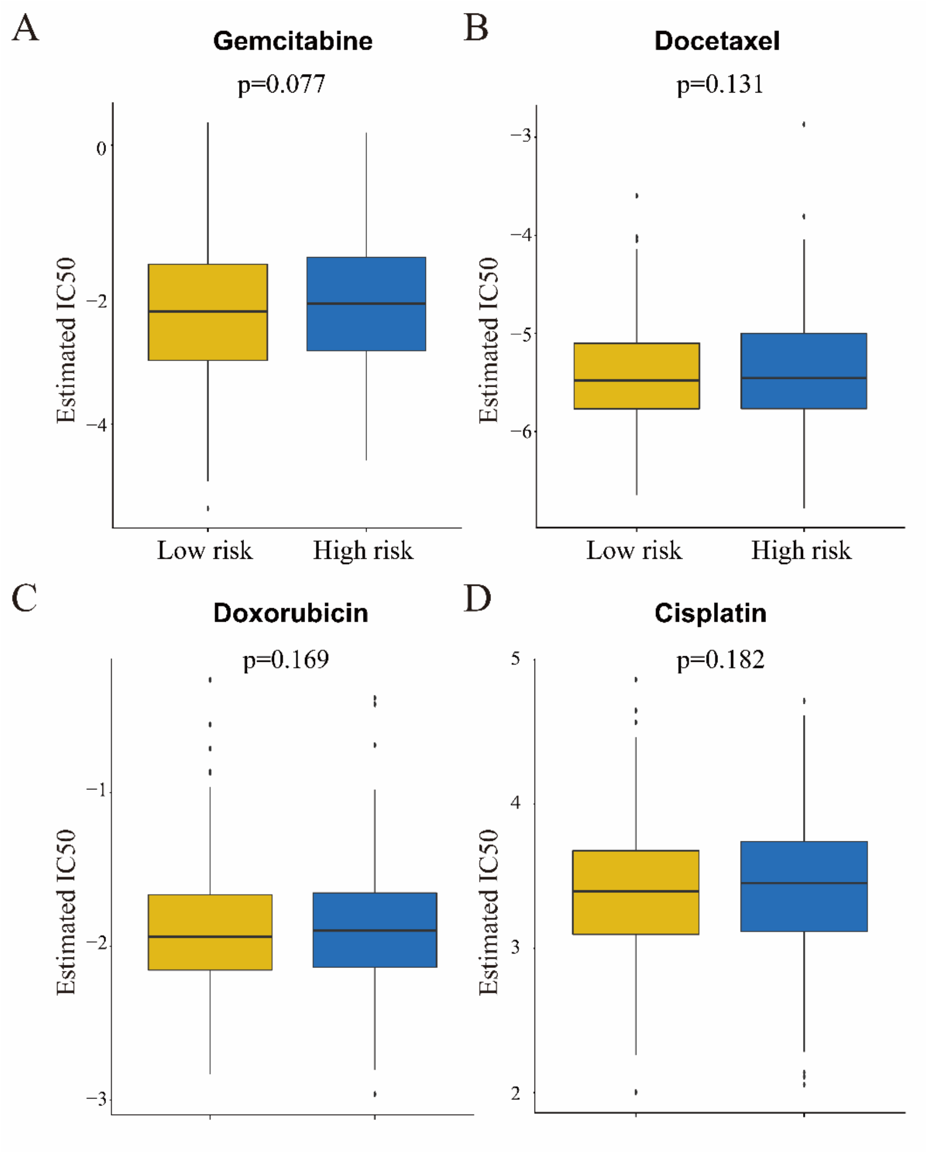

Supplement Figure 6. Estimated IC50 values indicate the efficiency of chemotherapy by gemcitabine, docetaxel, doxorubicin and cisplatin in both groups.

Supplement Table 1. Clinical characteristics of all SOC subjects in TCGA database.

Characteristics	All subjects (n=564)
**Age**	
>50 years	191
≤50 years	373
**FIGO stage**	
I	15
II	27
III	434
IV	84
Unknown	4
**Living status**	
Living	225
Death	339
**Grade**	
G1	6
G2	69
G3	475
G4	1
GB	1
GX	9
Unknown	3
**Residual tumor size**	
No Macroscopic (<1mm)	119
1-10mm	239
11-20mm	38
>20mm	102
unknown	66
**Lymphatic invasion**	
Yes	135
No	82
Unknown	347

Supplement Table 2. Clinical characteristics of SOC subjects in training cohort and test cohort.

characteristics	Training cohort (n=282)	Test cohort (n=282)	*p* value
age			0.4104
>50 years	103	88	
≤50 years	179	194	
FIGO stage			
I	11	4	0.6542
II	12	15	
III	221	213	
IV	37	47	
Unknown	1	3	
Grade stage			0.6890
G1	0	6	
G2	32	37	
G3	244	231	
G4	0	1	
GB	0	1	
GX	5	4	
Unknown	1	2	
Living status			0.6393
Death	175	164	
Living	107	118	

Supplement Table 3. All TFs were selected by univariant cox regression.

Characteristics	Hazard.Ratio	95%CI	P.value
SPDEF	0.63	0.48-0.83	0.000788
CREB3	0.68	0.54-0.85	0.000884
TEAD1	1.64	1.22-2.2	0.000965
IRF4	0.2	0.07-0.56	0.002255
TFAM	0.64	0.48-0.86	0.003081
ZHX3	2.32	1.31-4.12	0.003938
FOXK2	0.68	0.53-0.89	0.004322
LHX2	1.28	1.08-1.53	0.004367
FOXJ1	0.82	0.72-0.94	0.004485
RB1	1.75	1.19-2.58	0.004535
ZNF8	1.55	1.14-2.1	0.00468
ZXDB	0.23	0.08-0.63	0.004759
TRIM38	0.73	0.59-0.91	0.005132
STAT2	3.46	1.44-8.3	0.005441
ABT1	0.73	0.58-0.92	0.006893
KEAP1	0.73	0.58-0.92	0.007243
MAZ	0.54	0.34-0.85	0.00777
TRIM27	0.72	0.56-0.92	0.008753
CBX5	0.75	0.61-0.93	0.009149
CSRP1	0.74	0.59-0.93	0.010307
ZNF124	0.79	0.66-0.95	0.010308
TBX2	0.73	0.57-0.93	0.010313
ZNF76	0.57	0.37-0.88	0.011028
FOXG1	1.6	1.11-2.3	0.012214
BLOC1S1	0.71	0.55-0.93	0.01272
NFX1	0.61	0.41-0.9	0.01316
TBC1D10B	0.66	0.48-0.92	0.013304
GTF3C5	0.59	0.38-0.9	0.014672
KLHL21	1.27	1.05-1.54	0.01561
TRIM15	0.24	0.08-0.77	0.016024
TRIM26	0.74	0.57-0.95	0.017302
HIC1	3.12	1.22-7.95	0.017319
ZNF175	1.45	1.07-1.96	0.017349
ZNF672	0.73	0.57-0.95	0.018434
SOX4	0.79	0.65-0.96	0.018796
PBX1	0.78	0.64-0.96	0.019287
HOXB3	2.1	1.13-3.91	0.019315
ZNF184	0.81	0.68-0.97	0.019935
PRDM2	1.69	1.08-2.65	0.021206
KLF1	0.23	0.06-0.8	0.02127

GTF2H4	0.74	0.57-0.96	0.021875
ST18	4.18	1.23-14.25	0.022057
ZNF165	0.8	0.66-0.97	0.022718
AFF2	0.21	0.05-0.81	0.02367
ZNF141	0.59	0.38-0.93	0.024174
ZBTB7A	2.43	1.12-5.28	0.024489
MLLT3	0.77	0.62-0.97	0.024778
SP1	1.96	1.09-3.51	0.024903
CBX7	1.28	1.03-1.59	0.025606
ZNF32	0.73	0.56-0.96	0.026052
HOXD11	1.61	1.06-2.44	0.026256
SUPT6H	1.47	1.04-2.08	0.027075
MLLT10	1.54	1.05-2.26	0.027698
SUB1	0.72	0.54-0.97	0.028507
SSX1	1.61	1.05-2.46	0.029208
REL	0.59	0.36-0.95	0.030842
POU2AF1	0.84	0.71-0.98	0.031096
ZNF12	1.27	1.02-1.58	0.031501
SMARCB1	0.76	0.6-0.98	0.032793
NR1D2	1.24	1.02-1.52	0.033117
RXRG	0.64	0.42-0.97	0.035154
ASCL3	2.84	1.07-7.56	0.036252
TRIP6	1.27	1.02-1.58	0.036295
SMARCAL1	0.72	0.52-0.98	0.036423
GATA2	1.34	1.02-1.75	0.036458
SOX21	3.26	1.07-9.89	0.037286
ALX1	1.36	1.02-1.81	0.037298
TULP1	2.6	1.06-6.41	0.03749
MEF2C	1.5	1.02-2.21	0.038051
BRD2	0.75	0.58-0.99	0.04043
MEIS2	0.9	0.82-1	0.040545
MED14	0.53	0.28-0.98	0.042218
ZNF395	0.8	0.64-0.99	0.042255
POU1F1	2.79	1.04-7.52	0.042336
TTLL4	0.77	0.6-0.99	0.042388
ZBTB32	2.31	1.03-5.2	0.042804
ZNF85	0.77	0.59-0.99	0.043905
TAF11	0.8	0.65-0.99	0.044364
CBX8	0.47	0.23-0.98	0.044475
ZFX	0.63	0.4-0.99	0.044646
RFX1	0.3	0.09-0.97	0.045183
CREBZF	0.54	0.3-0.99	0.046095
TP53	0.86	0.75-1	0.04622

ZNF136	0.82	0.67-1	0.047005
PMF1	0.77	0.6-1	0.049443
TIMELESS	0.83	0.69-1	0.049817
WT1	0.88	0.77-1	0.049994

Supplement Table 4. The different response to drugs commonly used to treating cancers in high risk and low risk group.

Anti-tumor drugs	*p* value
Metformin	3.65E-11
S.Trityl.L.cysteine	3.75E-07
Etoposide	3.33E-06
AKT.inhibitor.VIII	4.54E-06
Camptothecin	1.45E-05
Mitomycin.C	1.60E-05
GW843682X	2.08E-05
SL.0101.1	2.70E-05
EHT.1864	6.14E-05
BIBW2992	0.000157
A.443654	0.000172
ATRA	0.000731
TW.37	0.000844
Paclitaxel	0.002748
Salubrinal	0.004002
JW.7.52.1	0.005006
VX.680	0.005763
Methotrexate	0.007218
CGP.082996	0.029298
MS.275	0.032466
ABT.888	0.039649
AICAR	0.062312
Gemcitabine	0.076623
JNK.Inhibitor.VIII	0.077145
CCT018159	0.078046
BI.D1870	0.078878
GW.441756	0.095743
NSC.87877	0.100028
Erlotinib	0.128267
Doxorubicin	0.130995
Bortezomib	0.136457
Rapamycin	0.141261
RO.3306	0.154503
BMS.708163	0.156478
Docetaxel	0.168542

PF.4708671	0.169062
Cisplatin	0.181733
Epothilone.B	0.21721
Roscovitine	0.220107
Tipifarnib	0.251422
ZM.447439	0.28197
GSK.650394	0.290054
Lapatinib	0.362927
Cytarabine	0.367007
CGP.60474	0.38071
Vinblastine	0.406348
KU.55933	0.410563
BIRB.0796	0.417006
Z.LLNle.CHO	0.423471
MG.132	0.465304
Sorafenib	0.540238
Nutlin.3a	0.579765
ABT.263	0.632799
KIN001.135	0.647681
GNF.2	0.652848
BI.2536	0.657607
MK.2206	0.666486
AZD.2281	0.671732
AZD6244	0.730632
IPA.3	0.762691
Vinorelbine	0.77107
X681640	0.774498
Gefitinib	0.780199
Bleomycin	0.781573
CI.1040	0.785213
Parthenolide	0.818131
SB590885	0.829107
PHA.665752	0.835331
WO2009093972	0.842529
GDC0941	0.859086
Bicalutamide	0.88498
Sunitinib	0.904433
BMS.509744	0.907724
Bryostatin.1	0.917801
PD.0325901	0.923005
Thapsigargin	0.937752
AG.014699	0.938634
CMK	0.941644

LFM.A13	0.950303
Shikonin	0.961616
DMOG	0.974511
Vorinostat	0.977263
Cyclopamine	0.979146
BAY.61.3606	0.980112
JNK.9L	0.982806
AZD8055	0.985451
PF.02341066	0.986378
RDEA119	0.986646
GDC.0449	0.988798
AZD6482	0.99216
Elesclomol	0.992926
AZD7762	0.994797
XMD8.85	0.995998
Obatoclax.Mesylate	0.996266
BMS.536924	0.997123
AS601245	0.997217
BMS.754807	0.997994
CEP.701	0.998137
VX.702	0.998198
QS11	0.998701
Bosutinib	0.99877
AUY922	0.999127
OSI.906	0.999146
NU.7441	0.999328
Lenalidomide	0.999412
AZ628	0.999509
PD.0332991	0.999585
BX.795	0.999744
NVP.BEZ235	0.99978
CCT007093	0.999824
PD.173074	0.999861
Nilotinib	0.999866
AMG.706	0.999893
X17.AAG	0.999933
SB.216763	0.999937
JNJ.26854165	0.999949
PAC.1	0.999959
Axitinib	0.999965
Temsirolimus	0.999979
Embelin	0.99998
WZ.1.84	0.999987

A.770041	0.999994
Pazopanib	0.999996
FH535	0.999996
FTI.277	0.999998
PF.562271	0.999999
Imatinib	1
NVP.TAE684	1
Dasatinib	1
PLX4720	1
GSK269962A	1
Bexarotene	1
AZD.0530	1
WH.4.023	1
AP.24534	1
Midostaurin	1
Pyrimethamine	1
CHIR.99021	1
